# Consequences of nano and microplastic exposure in rodent models: the known and unknown

**DOI:** 10.1186/s12989-022-00473-y

**Published:** 2022-04-21

**Authors:** Walison Augusto da Silva Brito, Fiona Mutter, Kristian Wende, Alessandra Lourenco Cecchini, Anke Schmidt, Sander Bekeschus

**Affiliations:** 1 Leibniz Institute for Plasma Science and Technology (INP), ZIK Plasmatis, Felix-Hausdorff-Str. 2, Greifswald, Germany; 2 Department of General Pathology, State University of Londrina, Rodovia Celso Garcia Cid, Londrina, Brazil

**Keywords:** Animal, Environment, Mice, Particles, Polymers, Rats, Toxicity

## Abstract

The ubiquitous nature of micro- (MP) and nanoplastics (NP) is a growing environmental concern. However, their potential impact on human health remains unknown. Research increasingly focused on using rodent models to understand the effects of exposure to individual plastic polymers. In vivo data showed critical exposure effects depending on particle size, polymer, shape, charge, concentration, and exposure routes. Those effects included local inflammation, oxidative stress, and metabolic disruption, leading to gastrointestinal toxicity, hepatotoxicity, reproduction disorders, and neurotoxic effects. This review distillates the current knowledge regarding rodent models exposed to MP and NP with different experimental designs assessing biodistribution, bioaccumulation, and biological responses. Rodents exposed to MP and NP showed particle accumulation in several tissues. Critical responses included local inflammation and oxidative stress, leading to microbiota dysbiosis, metabolic, hepatic, and reproductive disorders, and diseases exacerbation. Most studies used MP and NP commercially provided and doses higher than found in environmental exposure. Hence, standardized sampling techniques and improved characterization of environmental MP and NP are needed and may help in toxicity assessments of relevant particle mixtures, filling knowledge gaps in the literature.

## Introduction

Plastic debris is a growing environmental concern. In 2019, 368 million tons of plastic were produced globally [1]. Furthermore, pandemic-related single-use plastics (i.e., surgical masks) have worsened the scenario [2]. Despite recycling initiatives and legislation to ban single-use plastics, different plastic particles have been found in oceans, fresh water and agricultural systems, urban environments, the atmosphere, and remote areas such as the Mount Everest [3–5]. Small plastic particles are defined as microplastics (MP) (less than 5 mm diameter) and nanoplastics (NP) (less than 100 nm) [6, 7] and can vary in size, shape, type of polymer, and concentration [1, 3, 8, 9]. Regarding the sources, these are either deliberately manufactured (primary MP/NP) or derived from larger plastics during environmental exposure such as UV irradiation, mechanical abrasion, or microbial degradation (secondary MP/NP) [8].

Plastic particles are far-reaching and a multifaceted problem. The focus is not only on food [10, 11] or aquatic systems [4, 7, 8] as primary sources of plastic exposure but also on its epidemiological consequences [9, 12–14]. Small volume but large surface area facilitates chemical reactions with body fluids and tissues in direct contact with particle surfaces. These particles are of particular concern due to their persistence, bioaccumulation in the food chain and in wildlife destined for human consumption, potential toxicity, and ability to act as vectors for pathogens and co-pollutants [9, 12]. Marine organisms have also presented toxic effects of MP and NP exposure, depending on the type of organism, ultimately affecting bioaccumulation, metabolic changes, inflammation, reproduction effects, behavior, and ecosystem interactions [8, 15]. In addition, fish exposed to NP by environmentally relevant exposure route (contaminated prey ingestion) showed NP accumulated in different fish tissues and affected innate immune gene signatures. This exposure may compromise their ability to survive in nature [16].

Humans are exposed either directly to MP and NP in drinking water, sea salt, and the atmosphere or indirectly through the food chain [8–11]. Debris from plastic prosthetic implants is also a source of exposure to MP and NP in humans [9]. Moreover, the accumulation of particles in all trophic levels may expose humans to more particles in food sources [10, 13]. In a recent systematic review about MP content in American food sources, a caloric intake-based calculation was used to estimate human ingestion of a large number of particles (> 50,000) per year, significantly rising if drinking bottled water was included [17]. Such studies are necessary to raise public awareness about the constant uptake of plastic into the human body. It remains a matter of debate, however, which types of particles or their size or cargo as well as location may be critical in driving specific health-related conditions and diseases.

Continuous sources of less-concentrated MP (food containers and drinking water) are also a concern. Regulators (EFSA/WHO) state that MP and NP exposure in humans present few adverse effects, although this statement may be due to little evidence rather than a lack of effects. Preliminary signs of harm are still arising. The precautionary principle recommends and supports initiatives to develop better analytical methods before concluding that MP and NP exposure is entirely safe after all [18].

Current estimations of plastic particle exposure in humans are limited due to the lack of an established method to provide non-destructive evidence of MP and NP presence in tissue [10]. Ultra-thin sections of tissue, often used in medical research, cannot clarify the possible involvement of plastic in disease processes, as plastic is technically challenging to identify due to its small size and chemical inertness. Assessment of MP and NP exposure in rodent models offers a valuable tool to assess health risk of plastic exposure to animals and parallel it to humans. In addition, many established rodent models of human diseases offer the possibility to assess the sensitivity of specific pathologies to MP and NP exposure. We review recent findings from MP exposure within in vivo rodents model systems intending to give an outlook on them beyond the highlighted gastrointestinal and respiratory tract possible effects and fill knowledge gaps within other systems as well.

## Searching methods

In this scoping review, we used different combinations of keywords in the Google Scholar database between 2001 and 2021: "microplastics"; "nanoplastics"; "exposure"; "oral administration"; "inhalation"; "rodents"; "mice"; "rats"; "accumulation"; "toxicity" and "toxic effects". Inclusion criteria were original studies published in peer-reviewed journals and performed by exposing rodents (mice and rats) to MP and NP, assessing the accumulation of particles in tissues and/or toxic effects. With that, 31 original studies were included and described in Table 1. The remaining manuscripts were included as complementary information.

**Table 1:** Assessment of plastic particles in rodent models

Ref	Model	Polymer	Size	Route	Controls	Dose	Duration	Accumulation/effects
Oral administration								
Liang et al. [23]	C57BL/6 mice	PS (pristine or fluorescent)	50, 500, and 5000 nm, alone or in mixture	Oral gavage	Double distilled water	2.5, 25, 50, 125, 250, 500 mg/kg bw	Single dose, 24 h. Daily, 28 days	Single-dose: bioaccumulation over time in intestines (IVIS). Bioavailability in other organs was size-dependent, with small particles in the gut, liver, spleen, kidneys, heart, lungs, testis, epididymis, brain, blood, ovaries, and uterus. Larger particles were found in the blood and gut but not in other organs. They have altered mucus production in the gut. Co-exposure with different sizes increased biodistribution in organs and increased ROS generation, epithelium apoptosis, and permeability in the intestines. Antioxidant pre-treatment was able to reverse the effects. The 28-day repeated exposure model showed the same effects
Amereh et al. [30]	Wistar rats	PS	25 and 50 nm as mixture	Oral gavage	Distilled water	1, 3, 6, 10 mg/kg bw-day	Daily, 35 days	Decreased serum testosterone, luteinizing hormone, follicle-stimulating hormone, altered sperm concentration, motility, morphology, DNA damage. Histopathological signs of testes atrophy and degeneration and particles accumulation
Deng et al. [35]	CD-1 mice	PE (coated with phthalate esters)	0.4 to 3.2 µm	Oral gavage	Pure water, phthalate esters, virgin MP	100 mg/kg bw	Daily, 30 days	Phthalate ester accumulation in gut > liver > testes. Testes: reduced sperm count and viability, increased oxidative stress (SOD, MDA), increased spermatogenesis disorder markers LDH and ACP by MP contaminated with phthalate ester
Stock et al. [38]	C57BL/6 HOTT reporter mice	PS (fluorescent carboxyl or sulphate coated)	1, 4, and 10 µm in mixture	Oral gavage	0.5% (m/v) carboxymethylcellulose	1.25 – 34 mg/kg bw adjusted for surface area	Three times a week, 28 days	No effects observed: no Hmox1 reporter response or changes in body or organ weights and low intestinal particle retention. No pathological changes were measured by histology, and very low concentrations of particles in the intestines
Deng et al. [41]	ICR mice	PS (pristine or fluorescent)	5 and 20 µm	Oral gavage	Water	0.01–0.5 mg/day	Up to 28 days	Accumulation in gut, liver, and kidney. Liver inflammation, hepatic lipid accumulation, oxidative stress, decreased AChE activity, altered lipid profile, and impairment of energy metabolism (reduction in ATP levels)
Li et al. [48]	C57BL/6 mice	PE	10–150 µm	Diet	Basal feed with no special preparation	6, 60, and 600 µg/day, adjusted for 3 g consumption/animal	35 days	Increased IL-1α in serum and different serum cytokine profiles depending on concentration. Small intestinal inflammation: increased TLR4, AP-1, and IRF5 protein (IF) and increased microbial diversity and abundance from fecal samples at the highest concentration of MP
Ding et al. [46]	SPF grade C57BL/6 mice	PS (fluorescent)	60 nm	Oral gavage	Double distilled water	50 µg/mL (500 µL)	Single-dose, 3 days	Particle accumulation in the stomach, intestines, and liver. No accumulation was observed in the heart, spleen, and lungs
Jin et al. [49]	ICR mice	PS (pristine or fluorescent)	5 µm	Drinking water	Reverse osmosis pure water	100 and 1000 µg/L	42 days	Accumulation in the gut was followed by gut microbiota dysbiosis and decreased mucous secretion. Intestinal barrier dysfunction. Significant increase in hepatic total bile acid (ns increases in serum) and altered bile acid metabolites. Altered amino acid metabolism: increased serum arginine, tyrosine, and succinylacetone
Luo et al. [50]	ICR mice	PS	5 µm	Drinking water	Water	100 and 1000 µg/L	During pregnancy and lactation (about 6 weeks)	Altered serum and hepatic metabolic markers; different levels of genes related to glycolipids and energy metabolism in dams, F1 and F2 offspring. No influences on F1 and F2 growth rate. Dams: hepatic ballooning degeneration. Altered gut microbiota and decreased mucus secretion. F1: altered serum metabolites. Adult female F1: lipid accumulation in the liver
Luo et al. [51]	ICR mice	PS	0.5 and 5 µm	Drinking water	Water	100 and 1000 µg/L	During pregnancy (about 6 weeks)	Increased risk of fatty acid metabolism disruption in offspring: In both sexes, 5 µm particle exposure reduced β-oxidation and fatty acid synthesis. Amino acid metabolism is reduced in females
Walczak et al. [52]	Fischer 344 rats	PS (fluorescent non-coated, aminated and carboxyl-modified)	50 nm	Oral gavage	Deionized water	125 mg/kg bw	Single dose, 6 h	All particles were observed in the lung, heart, kidneys, brain, stomach, and intestines. Negative NP was also in the liver. Estimated bioavailability: from 0.2 to 1.7%. No histopathological changes
Li et al. [69]	C57BL/6 mice	PS (fluorescent)	5 µm	Drinking water	Reverse osmosis water	20 mg/kg/day bw	30 days	Particle accumulation in the liver. Vacuolar degeneration, chronic inflammatory infiltration, and hepatocellular edema (histologically). Increased IL-1β and TNF-α mRNA (hepatic). Signs of apoptosis (TEM). Increased Nrf2 and Keap1 hepatic protein. Liver oxidative stress: decreased SOD and GSH, increased MDA
Deng et al. [70]	CD-1 mice	Suspended PE and PS in organic flame retardants (OFR)	0.5 to 1.0 µm	Drinking water	Water	2 mg/L (PE or PS) in 10 or 100 µg/L (OFR in aqueous solution)	90 days	Accumulation in liver and gut, with local inflammation and lipid droplets (H&E). Hepatic oxidative stress and LDH increased in MP + OFR, decreased AChE in the brain, and altered metabolomics in serum and liver
Jin et al. [71]	BALB/C mice	PS (fluorescent)	0.5, 4, and 10 µm	Oral gavage	Double distilled water	10 mg/mL	Daily, 28 days	Testicular accumulation followed by local inflammation. Reduced sperm quality and testosterone levels. Disruption of blood-testis barrier and disordered arrangement of spermatogenic cells with the presence of multinucleated gonocytes (H&E)
Lu et al. [79]	ICR mice	PS	0.5 and 50 µm	Drinking water	Reverse osmosis water	100 and 1000 µg/L	35 days	Decreased body, liver, and epididymis fat weights. Decreased mucus secretion in the gut. Altered biochemical serum markers. Changes in microbiota, hepatic lipid profile, and expression of some genes related to lipid metabolism decreased triglyceride synthesis markers mRNA in fat tissue
Silva et al. [84]	Swiss mice	PUR	250 nm	Oral gavage and IP	0.9% NaCl	2, 5, and 10 mg/kg bw	10 days	Oral gavage: increased visceral fat accumulation, glomerular atrophy, and increased serum TNF-α and ALP. IP: glomerular necrosis and inflammatory infiltrate in adipose tissue on the high dose. Both administration routes: lung inflammation, liver vascular congestion, and hepatocytes vacuolization. Increased ALT levels and serum IL-6
Zheng et al. [85]	C57BL/6 mice	PS	5 μm	Drinking water	Distilled water	500 µg/L	28 days	Exacerbated acute colitis model: increased intestinal permeability, lipid and liver metabolites disruption, triglyceride accumulation, and lipid peroxidation in the liver. Increased serum IL-1β, TNF-α, and INF-γ in mice exposed only to MP. In addition, MP exacerbated serum cytokines in the colitis model
Xie et al. [87]	BALB/c	PS	5.0–5.9 μm	Oral gavage	0.9% NaCl	0.01, 0.1 and 1 mg/d or 1 mg/d + NAC or p38 MAPK inhibitor	Daily, 42 days	Decreased sperm number, motility, metabolism markers LDH and SDH, serum testosterone, and GSH. Increased sperm deformity rate, ROS, MDA, apoptosis, and pro-inflammatory cytokines (IL-1β, IL-6, and TNF-α). Rescued by N-acetylcysteine and SB203580
Hou et al. [88]	ICR mice	PS	5 µm	Drinking water	Water	100, 1000 and 10,000 µg/L	35 days	Sperm count decreased, and deformities increased. Disordered arrangement of spermatic cells. Increased NF-κB, IL-1β, IL-6, and testicular apoptosis. Decreased HO-1 protein and Nrf2 protein and mRNA
Hou et al. [90]	Wistar rats	PS	0.5 µm	Drinking water	Deionized water	0.015, 0.15 and 1.5 mg/kg/d	90 days	Increased thickness of granulosa layer with some thinner secondary follicles (H&E) and decreased number of growing follicles. Decreased antioxidant defenses (GPx, SOD, and CAT). Increased MDA in ovaries, and NLRP3 and caspase-1 in ovarian granulosa cells (high dose). IL-1β and IL-18 increased, and anti-Müllerian hormone decreased
An et al. [91]	Wistar rats	PS	0.5 µm	Drinking water	Water	0.015, 0.15 and 1.5 mg/d	90 days	Decreased number and volume of growing follicles and ovary fibrosis in high concentration. Decreased anti-Müllerian hormone and decreased ovarian reserve capacity. Increased MDA and decreased antioxidant enzymes (SOD, CAT, GPx). Increased apoptosis, Wnt, and TGF-β in ovaries
Park et al. [93]	ICR mice	PE (containing surface modification with acid and hydroxyl groups)	40 to 48 µm	Oral gavage	Water	3.75, 15 and 60 mg/kg body weight	Daily, 90 days, some females exposed more than 21 days (lactation period)	90 days exposed males: decreased body weight gain, changes in hematological parameters. 90 days exposed females: altered hematological parameters and spleen immune response parameters, and increased serum IgA. 90 days exposed mice: hypertrophy/hyperplasia of stomachs mucosa. No adverse symptoms were observed in dams during gestation or lactation. Pups: altered sex ratio and growth rate, altered spleen immune response parameters
Rafiee et al. [98]	Wistar rats	PS	25 and 50 nm	Drinking water	Distilled water, dispersing reagent (surrounding medium)	1, 3, 6, 10 mg/kg body weight	Daily, 5 weeks	No effects observed: no differences in body weight. Neurobehavioral assessment alone. No cognitive changes
Li et al. [100]	Wistar rats	PS	0.5 µm	Drinking water	Deionized water	0.5, 5 and 50 mg/L	Daily, 90 days	Myocardium vascular congestion and accumulation of MP. Thinner and ruptured tissue in high dose followed by increased serum cardiac damage markers CK-MB and Troponin I. Increased MDA and reduced antioxidant enzymes in the heart. Increased myocardium apoptosis and fibrosis mediated by Wnt/β-catenin pathway activation
Amereh et al. [101]	Wistar rats	PS	25 and 50 nm mixture	Oral gavage	Distilled water	1, 3, 6 and 10 mg/kg body weight/day	Daily, 5 weeks	There were no effects on T3 and T4 hormones in serum; however, circulating active forms of thyroid hormones (FT3 and FT4) were decreased in rats. Increased TSH levels in high-dose. Changes in cholesterol serum markers and increased levels of liver damage markers (ALT and AST)
Inhalation/airways								
Eyles et al. [27]	BALB/c mice	Scandium-46 labelled styrene-divinyl benzene	7 µm	Intranasal instillation	Absent control group	0.250 mg (47.5 kBq) in 50 or 10 μl PBS	24 days	50 µL dose: substantial bronchopulmonary deposition, accumulation on liver and spleen. 10 µL dose: accumulation in nasopharyngeal regions only
Lim et al. [58]	SD rats	PS	0.1 µm	Inhalation	Fresh air control	0.68 × 10^5^, 1.38 × 10^5^ and 2.82 × 10^5^ particles/cm^3^	6 h each day, 5 days/week for 14 days (Modified OECD TG 412)	Serum AST and lung inspiratory time decreased in males. Respiratory frequency increased and inspiratory/expiratory time decreased in females. In females, reduced leukocytes count. Inflammatory markers: TGF-β and TNF-α increased in lung dose-dependently in both sexes. No changes in body weight or food consumption. No concentration–response was observed
Fournier et al. [59]	SD rats	PS (fluorescent)	20 nm	Intratra-cheal instilla-tion	0.9% NaCl	2.64 × 10^14^ particles	24 h	Accumulation in maternal lungs, heart, and spleen. Fetal liver, lungs, heat kidney, and brain. Significantly lower fetal and placental weights when adjusted for litter size variation. No differences in maternal weight or number of fetuses per litter
Other routes								
Estrela et al. [22]	Swiss mice	PS (fluorescent) and/or ZnO	PS NP: 23 nm ZnO: 69 nm	IP	Water	14.6 ng/kg	3 days	In separate, both particles induced cognitive impairment, redox imbalance (increased nitric oxide levels and thiobarbituric acid reactive species), and suppressed acetylcholinesterase activity. Systemic DNA damage was observed in separate and combined injections of particles
Kaga et al. [24]	Athymic nude mice	Radiolabelled PEGylated PS	Spherical: 21 and 33 nm, rod-like: 37 nm diamet., 350–500 nm length, worm-like: 45 nm diamet., 1–2 μm length	IV	Absent control group	0.1 mg in 50 µL PBS (2 mg/mL)	48 h	All particles accumulated in the liver, spleen, kidneys, heart, lungs, pancreas, thigh muscle, and tumor with different biodistribution
Hu et al. [94]	C57BL/6-mated BALB/c mice	PS	10 µm	IP	0.9% NaCl	250 µg in 200 µL saline	Pregnant mice on embryonic days 5.5 and 7.5	Increased embryo resorption rate and decreased number and diameter of uterine arterioles in the placenta of MP. Decreased leukocytes in blood, spleen, and placenta of dams. Decreased NK cells and macrophages in the placenta. Changes in macrophages polarization favoring M2-subtype, increased T CD4 + cells in the placenta, and changed cytokines secretion
Nie et al. [95]	ICR mice	PS	60 and 900 nm	IV	0.9% NaCl	300 µg	Pregnant mice on embryonic days 8, 9,10 and 15	No effects on number of embryos. Decreased body weight of embryos. 60 nm NP: decreased placental diameter, extravasation in fetus and placenta

PS, polystyrene; IVIS, in vivo image system; bw, body weight; MP, microplastics; ACP, acid phosphatase; HOTT, Heme-oxygenase triple transgenic; Hmox1, heme oxygenase-1; PE, polyethylene; IF, immunofluorescence; NP, nanoplastics; TEM, transmission electron microscopy; OFR, organic flames retardants; PUR, polyurethane; SDH, succinate dehydrogenase; SB203580, p38 MAPK inhibitor; CAT, catalase; ZnO, zinc oxide nanoparticles; IP, intraperitoneal; PEG, polyethylene glycol; IV, intravenous

## Discussion

### Plastics utilized in rodent models

Plastics are synthetic polymers derived from fossil fuels or biomass. The most common polymers produced globally include polyethylene terephthalate (PET), polyethylene (PE), polyvinyl chloride (PVC), polypropylene (PP), polystyrene (PS), and polyurethane (PUR) [19]. Heterogeneous plastic mixtures contaminate environmental sources such as water [20, 21], in which environmental fragmentation and degradation may hinder their classification, generating products with different shapes, sizes, chemical compositions, and densities [14]. However, most rodent studies used one plastic entity (Table 1) and not with heterogeneous mixtures as found in the environment.

Commercially available particles are uniform spheres with pristine or functionalized surfaces. Despite the characterization of exposure effects of a particular polymer, commercial specifications do not reflect environmental exposure accurately [14]. To this end, Estrela and colleagues assessed acute exposure to the combination of zinc oxide nanoparticles and PS NP in mice [22]. Although pathophysiological changes were observed from exposure to PS NP (Table 1), no additive or synergistic effects were observed when administered in combination. Moreover, Liang and colleagues found that MP and NP mixtures with different sizes facilitate biodistribution in mice's tissues [23].

Secondary MP and NP exhibit diverse shapes and surfaces from environmental weathering that may influence biodistribution. For example, an assessment of tritiated polyethylene glycol (PEG)ylated PS in a tumor model nude mouse highlighted the accumulation of rod/worm-like particles in the liver and spleen compared with retention of small spherical particles in tumor masses [24]. However, further work is needed to determine the effects of polydisperse environmental secondary particles. In addition, the development of improved sampling methods to accurately characterize 'natural' particles is necessary [20, 21].

According to our literature review, label-free determination of plastic in cells and human-relevant systems has not yet been successful, although innovative microscopic or spectroscopic methods (e.g., UV light spectrum, infrared light spectrum, and Raman spectrum) are still emerging [25]. Radio-labeled plastic particles are used to include quantitative whole-body radiography in marine organisms and determine the mass balance in mice [24, 26, 27]. Fluorescently-labeled MP and NP facilitate direct quantification of bioaccumulation in tissues. Also, many commercial particles are produced with internalized fluorescence, avoiding dye-specific interactions on the particle surface. Nonetheless, possible effects of label leaching over time must be considered [28, 29].

Quantifying particle deposition within tissues helps determine whether responses are due to direct interactions with particles or indirect secondary effects [28, 29]. Monitoring labeled polymers non-invasively offer the potential for real-time measurements. For instance, Amereh and colleagues observed the accumulation of a mixture between 25 and 50 nm polystyrene particles in testes of Wistar rats using in vivo imaging system (IVIS) [30]. Another study using IVIS showed accumulation over time in the intestines of mice exposed to MP and NP [23]. However, longitudinal monitoring of fluorescent probes is hampered in deep tissues by signal penetration and tissue autofluorescence. Also, due to the low resolution, positive fluorescent signals are likely to be aggregates rather than being dispersed particles. Those difficulties may justify the observation of particle fluorescence only in peripheral tissues.

Plastic contaminants should not be viewed as isolated particles as several organic and non-organic molecules can adhere to them. Proteins can, for example, form a protein corona around particles [31, 32]. However, it is unclear whether these are human-relevant proteins and their effect. Other toxic molecules can also bind to plastic (some of them already during the manufacture of plastic products) and are slowly released later into the environment or the body [33]. Moreover, plastic binds to lipids or changes their composition in cell membranes, which may occur in freshwater algae [34]. However, we did not find any information on such phenomena in rodents or human-relevant systems.

Due to synthetic production and environmental degradation, plastics are in close contact with several types of additives and pollutants, such as phthalates, bisphenol analogs, surfactants, and pigments, all associated with potential toxic effects [14]. For example, Deng and colleagues demonstrated phthalate ester accumulation in the gut, liver, and testes following exposure to PE MP by oral gavage [35]. Moreover, several chemicals can act as endocrine disruptors, i.e., affecting hormones pathways or acting as pseudo-hormones themselves [36, 37].

In summary, improved sampling methods to determine the most common environmental particle properties will help to streamline the systematic characterization of the effects of individual polymers of different shapes, sizes, and associated coronas. In addition, the experimental utilization of heterogeneous mixtures of particle combinations and environmental plastic samples may contribute to a better understanding of the potential additive effects and effects of chemicals that come as cargo with MP and NP exposure.

#### Dosage

The environmental relevant dose of MP and NP exposure is heavily debated. Many studies use MP and NP concentrations far greater than current human exposure estimates (Table 1). Estimations are that human consumption of up to 0.06 mg/kg/day of particles occurs via drinking water [30]. Administration of a high single dose of particles followed by substantial recovery or constant exposure of concentrated particles is unlikely to reflect real-world scenarios. To this end, Stock and colleagues used a dosing regimen of PS MP at less than 34 mg/kg body weight thrice weekly for four weeks [38]. They found minimal particle uptake into intestinal tissue and no toxic effects.

Conversely, high concentrations reflect the combination of multiple exposure routes in nature [39] and emulate increases in microplastic pollution in the future. Current limitations in methods to detect MP and NP accurately hinder estimations of environmental concentrations unreliable [40]. Therefore, determining the threshold at which MP and NP exposure is associated with adverse events remains critical.

#### Polymer exposure routes

Oral ingestion of plastic and absorption via the gastrointestinal tract has so far been the focus of MP/NP research [38]. However, reports in which plastic particles sized up to 20 µm are ingested [41] do not seem comprehensible according to the assessment of the German Federal Institute for Risk Assessment (BfR) [42]. Although microparticles up to 150 µm can translocate across mammals' intestinal barriers [43], the absorption rate is below 0.3%. From the rate, mostly particles sized up to 10 µm should be able to penetrate all organs, including the brain, with unexplored consequences [44].

Low absorption of MP and NP through intestinal epithelium could be related to particles properties and efficiency of the mucus barrier to interact and maintain MP and NP in the intestinal lumen. By being maintained, MP and NP can be excreted in the feces or deposited, which may cause local irritation or release of toxic additives [44]. Also, MP and NP can be internalized by intestinal epithelium and be re-released into the intestinal lumen due to a turnover of approximately 3 days, thus not reaching the bloodstream [45]. Currently, some studies assume that toxic effects are expected in the digestive tract and liver due to continuous plastic accumulation (Table 1) [46, 47]. A murine model fed with PE particles showed increased inflammation in small intestines followed by changes in microbiota and increased systemic pro-inflammatory markers [48].

Another route for human exposure to MP and NP is drinking water, as plastic particles were detected in tap and bottled water [17]. Some studies used this administration route to expose rodents models to MP and NP (Table 1). However, water consumption was not assessed for particle intake calculations [49–51]. Additionally, this route is not appropriate for assessing buoyant polymers such as PP and PE and may be inefficient considering particle sedimentation over time for MP and NP suspensions. Another limitation of the oral uptake route (drinking water, diet, and oral gavage) might be bioavailability, which was estimated to range from 0.2 to 1.7% with different types of NP in vivo [52].

Plastic is not only absorbed by food through the digestive tract [53]. It can also be inhaled through fine air dust (e.g., abrasion from car tires or clothing [54, 55] and release chemical additives [56] once within the body [57]). Occupational diseases associated with textiles have been extensively reviewed [54]. Fragments and fibers are the most common forms of atmospheric MP and NP. However, estimations of human exposure levels are limited by the lack of sensitivity of current methods to detect small particles [5, 58].

Clearance of inhaled particles can be through mucociliary transport resulting in negligible deposition in airways or phagocytosis by alveolar macrophages or lymphatic transport [54]. MP and NP may avoid these mechanisms, accumulating in the lungs and entering systemic circulation [27, 58, 59]. Inhaled nanoparticles can also reach the central nervous system (CNS) through the olfactory bulb [60]. A recent 14-day repeat inhalation study in rats highlighted lung inflammation and decreased inspiratory rate following exposure to 100 nm PS particles [58]. Also, a single intratracheal dose during gestation resulted in maternal-to-fetal translocation of PS NP [59].

Topical exposure to MP and NP from microbeads in personal hygiene products and contaminated water may directly affect the skin. Epidermal cells exposed to MP and NP in vitro exhibited oxidative stress [61]. However, uptake across the outermost skin layer, the *stratum corneum*, is considered restricted to nanoparticles smaller than 100 nm [43]. Minimal uptake was observed following ex vivo administration of 20–200 µm fluorescent particles to pig ears both with and without compromised barrier function [62]. Particle weathering and aging may enhance topical uptake, as observed in mice with quantum dot nanoparticles [63]. To our knowledge, topical plastic exposure has not been extensively characterized in rodent models.

Various exposure routes have been utilized in animal models. Oral and inhalation routes are considered the main exposure routes in humans. The influence of a particular administration route on particle characteristics (e.g., accompanying corona or ability to release toxic chemicals) is not well understood.

### In vivo effects of polymer exposure

**Fig. 1:**
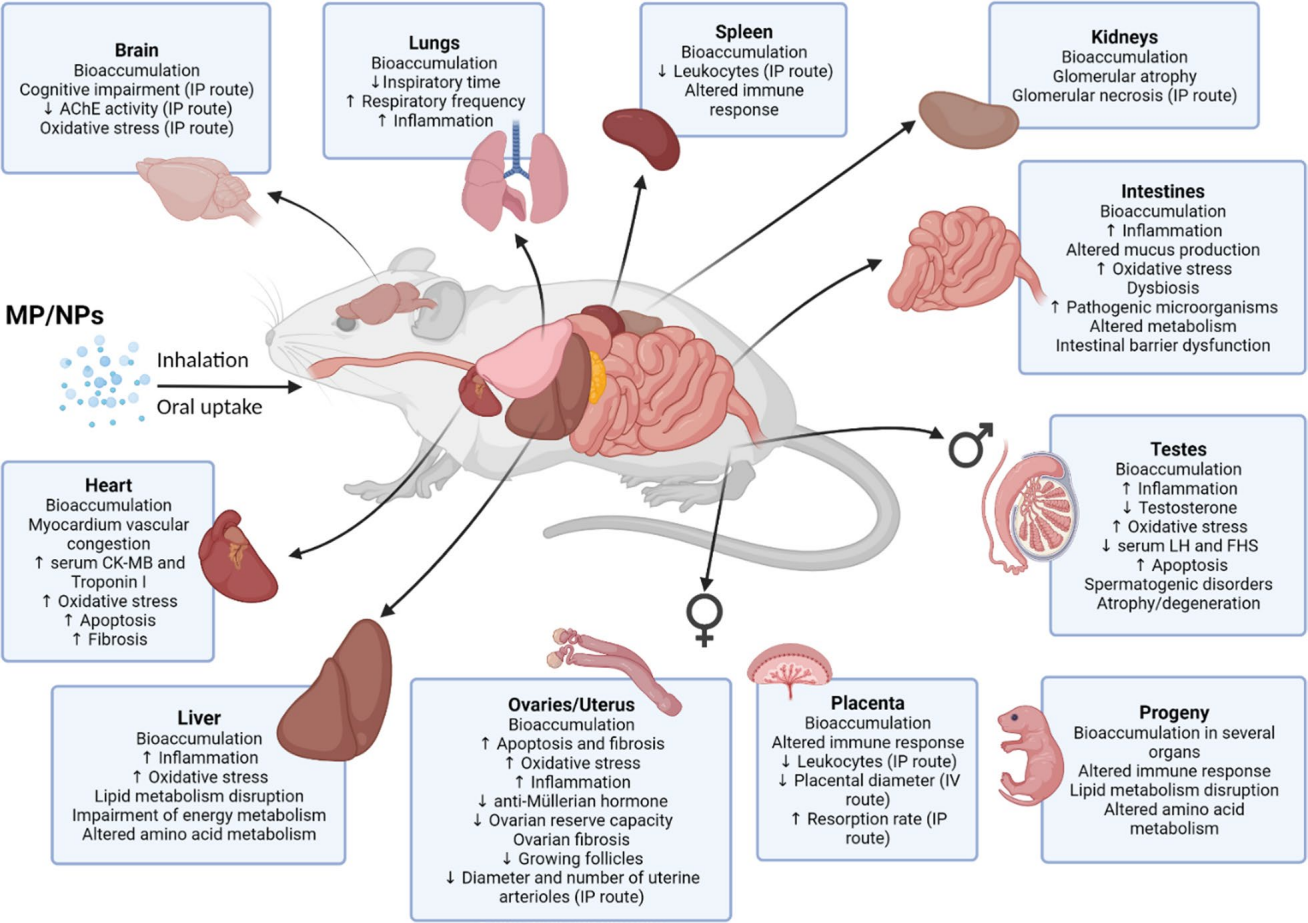
Biological effects observed in rodents exposed to MP and NP. *Abbreviations* MP/NPs, micro and nanoplastics; AChE, acetylcholinesterase; IP, intraperitoneal; LH, luteinizing hormone; FSH, follicle-stimulating hormone; IV, intravenous. Created with BioRender.com

Despite being considered chemically inert compared to plastic monomers, toxicity following MP and NP exposure was described (Fig. 1, Table 1). MP and NP toxicity may result from their persistent physical presence in tissues. Size-dependent effects have been demonstrated in vitro with PS spheres [61, 64]. Small and positively charged particles may have greater bioavailability in mammals [65]. Particle accumulation has been demonstrated in organs such as the liver, kidneys, brain, spleen, and reproductive organs (Fig. 1, Table 1), although it was independent of the functionalized surface coating in high concentration [52].

Disruption, penetration, absorption, and endocytosis mechanisms, which may be toxic [66], are currently being discussed as possible ways plastic particles can enter and interact with cells and tissues [67, 68]. Possible toxic consequences may not only be due to MP and NP exposure, as most commercially available particles used in studies in vivo are provided in aqueous suspensions with dispersant and conservant solvents. Walczak and collaborators centrifuged the particles for conservant and surfactant removal before usage, controlling possible effects found after exposure [52]. Thus, evaluating additional compounds as control groups and not only test vehicle solutions is essential.

#### Direct effects and underlying mechanisms

In mice exposed to fluorescently labeled particles, localized inflammation at the site of particle accumulation has been confirmed in the liver [41, 69, 70] and testes [71]. However, fluorescent dye leaching from MP and NP could also contribute to the exposure effects observed. Interestingly, few studies evaluated the fluorescent dye leaching of particles under conditions such as simulated gastric and intestinal fluids, and fluorescence leaching was negligible [23, 52]. Moreover, fluorescent MP and NP are mainly used only for bioaccumulation and biodistribution assessments into tissues, and non-fluorescent for toxicity evaluations [23, 41]. Exposure to non-fluorescent particles resulted in increased inflammation in primary absorption sites consistent with the exposure route, such as the gut [48] and lungs [58].

One proposed central mechanism for MP and NP toxicity is the induction of oxidative stress, which has been extensively observed in vitro [72]. However, another study found the opposite effect, a reduction of plastic-induced oxidative stress in cells in vitro [73]. In addition, some cell types can actively excrete plastic particles [64], possibly influencing the response to oxidative stress [74]. Mice exposed to drinking water with high concentrations of MP showed impaired antioxidant defenses, such as decreased superoxide dismutase (SOD) and glutathione (GSH) expression and increased malondialdehyde (MDA) formation (a product from lipid peroxidation). In addition, increased activity of the Nrf2/Keap1 pathway was observed, suggesting plastic-induced oxidative stress and its relation with inflammation in the tissue microenvironment [69].

Regarding additives and pollutants leached from plastic particles, mice exposed to MP and NP (PS and PE) by drinking water with organic flame retardants presented more pronounced oxidative stress in the liver [70]. Testes of mice exposed to oral gavage with PE coated with phthalate esters also showed oxidative stress responses [35]. However, these effects may be due to additives released in the solution and not to MP and NP exposure, as no information was provided regarding solutions stability over time or whether they were used as fresh preparations [35, 70]. Mice exposed to a single dose of MP and NP mixtures with different sizes by oral gavage showed increased ROS generation, intestinal epithelium apoptosis, and intestinal permeability, and pre-treatment with antioxidants reversed the effects [23].

Current studies do not indicate genotoxicity or mutagenicity of everyday plastics, as shown for PS [75]. In contrast, an in vitro study in human fibroblasts [76] and an investigation into the damage to cell-free DNA [77] indicated corresponding genotoxicity. However, other types of plastic and rodents models have hardly been investigated to confirm effects on a broader species scale.

#### Gastrointestinal toxicity

Plastic exposure in the intestines of mice induces local inflammation [48], alters microbiomes [78] especially favoring facultative pathogenic *S. aureus* strains [48], provokes metabolic dysfunction [49], influences liver lipid metabolism [79, 80], and modifies host–pathogen interactions [81]. Although these results seem relevant for humans [82], most effects occurred with high MP and NP doses in unspecific endpoints not simulating environmental conditions.

Changes to the intestinal microbiota contribute to metabolic disorders, including obesity and diseases such as colorectal carcinoma [82, 83]. Li and colleagues observed increased microbial load and diversity in fecal samples of mice fed with PE particles (600 µg/day for 35 days) [48]. Gut dysbiosis coincided with increased hepatic bile acid levels and altered serum bile- and amino acid-related metabolites in mice exposed to high concentrations of 5 µm PS MP (100 and 1000 μg/L) in drinking water for six weeks [49].

#### Hepatotoxicity

In response to oral exposure to MP and NP, multiple groups showed altered gut microbiome and disruption of serum and hepatic markers of amino acid synthesis and metabolism, energy, and lipid metabolism [49–51, 79], followed by liver inflammation [41, 69]. Hepatocellular edema and inflammatory cell infiltration were observed with increased hepatic IL-1β and TNF-α mRNA following exposure to 5 µm PS particles (20 mg/kg/day body weight) in drinking water for 30 days [69]. The extent of hepatotoxic insult was not sufficient to alter serum markers of liver function (alanine transaminase [ALT] and aspartate aminotransferase [AST]) after the exposure period. However, mice exposed to 250 nm PUR particles by oral gavage for 10 days showed increased serum ALT, alkaline phosphatase (ALP), IL-6, and TNF-α levels, followed by liver vascular congestion and hepatocytes vacuolization [84]. Accumulation quantification of fluorescent particles was hindered by extensive tissue autofluorescence, hampering to conclude whether the effects were associated with the presence of hepatic particles.

Stock and colleagues treated heme oxygenase-1 (HO-1) triple transgenic (HOTT) reporter mice with a mixture of 1, 4, and 10 µm PS particles by oral gavage [38]. These animals expressed a LacZ reporter sensitive to oxidative stress and inflammation. However, the study found no positive responses or pathological changes to the liver or other organs, possibly due to the low concentrations of particles (1.25–34.0 mg/kg body weight for particles mixture every 3 days for 28 days).

The liver is the primary site for lipid metabolism and is sensitive to pathologies such as nonalcoholic fatty liver disease (NAFLD) that manifest as an accumulation of fatty vesicles combined with elevated circulatory cholesterol and triglycerides [83]. Lipid disruption in response to MP/NP exposure in rodents has been observed by multiple groups [50, 51, 82, 85]. Luo and colleagues observed hepatic ballooning (characteristic of apoptosis), increased hepatic triglycerides, total cholesterol, and decreased PPARα and PPARγ mRNA in maternal mice after exposure to 5 µm PS MP (100 and 1000 µg/L) by drinking water during gestation and lactation [50]. Disrupted PPAR signaling and decreased hepatic triglycerides and total cholesterol were also observed in F1 offspring. The lipid-sensitive nuclear receptor PPARα regulates fatty acid catabolism and clearance and is thought to have anti-inflammatory effects (NF-κB suppression) [86]. Therefore, the extent of hepatic PPARα downregulation is predictive of NAFLD severity.

PPARγ is also downregulated during hepatic stellate cell activation, resulting in fibrosis [86]. At lower concentrations of 5 µm PS (500 µg/L), hepatic fatty vacuoles were observed in male C57BL/6 wild-type mice exposed to MP by drinking water for 28 days, without changes to hepatic triglyceride or PPARγ at the protein level [85]. This result indicates potential strain and/or gender differences, although a lack of water intake assessment may have resulted in different particle exposure between individuals. However, Lu and colleagues observed decreased liver weights and hepatic and circulatory levels of total cholesterol and triglycerides with downregulation of hepatic triglyceride synthesis in male mice exposed to 0.5 and 50 µm PS MP (100 and 1000 μg/L) by drinking water for 35 days. At the mRNA level, increased PPARα and decreased PPARγ expression were identified [79].

Changes in lipid metabolism are thought to be dependent on particle size. F1 offspring from dams exposed to 0.5 and 5 µm PS particles (100 and 1000 µg/L) in drinking water during gestation exhibited decreased hepatic total cholesterol and triglycerides in a particle dose- and size-dependent manner [51]. In addition, decreased PPARα hepatic mRNA expression was observed in groups exposed to 5 µm MP alone. Whether these effects are due to altered maternal metabolism, making offspring more susceptible to disease, or particles transferred to the fetus directly affecting the next generation remains unclear.

#### Reproductive dysfunction

MP and NP have been shown to accumulate in reproductive tissues [23, 30] and cross the placental barrier [59]. Accumulation of MP and NP in testes of rodents corresponded with histological changes followed by local inflammation and DNA damage in germ cells [30, 71, 87]. Also, rodents exposed to MP and NP by oral gavage showed decreased serum testosterone levels, a hormone essential for spermatogenic cells development [30, 71, 87]. These observed effects were alleviated in male mice treated with ROS scavenging compounds because oxidative stress was induced through p38 MAPK signaling pathway activation after MP exposure [87]. This pathway is also involved in inflammation, which could explain increased levels of pro-inflammatory cytokines in testes of mice exposed to MP and NP [71, 87, 88]. Additionally, mice exposed to MP by drinking water demonstrated increased NF-κB followed by decreased Nrf2 and HO-1 in testes, suggesting this increased pro-inflammatory profile may be due to reduced Nrf2/HO-1-mediated NF-κB inhibition pathways [88].

Plastic exposure of mice dams caused far-reaching effects on milk ingress [50] and generally metabolic syndromes [51] in first and second-generation offspring of the first and second generation, regardless of sex [89]. In ovaries, exposure to MP by drinking water for 90 days reduced the number and volume of growing follicles and anti-Müllerian hormone levels and induced oxidative stress in rats [90, 91]. In addition, oxidative stress triggered cell death mechanisms, inflammation [90], and fibrosis through Wnt/β-catenin pathway activation in ovaries [91]. Changes in the uterus due to plastic exposure were also observed [92], with altered number and gender ratio of offspring of parents exposed to PE MP by oral gavage during pregnancy. However, tendencies were not dose-dependent [93].

Exposure to PE MP in dams by oral gavage during pregnancy and lactation altered the development and number of T cells in spleens in offspring of both sexes. Also, the maturation of dendritic cells was inhibited in males and enhanced in female pups [93]. Furthermore, in an allogeneic mating murine model, pregnant mice exposed to PS MP by IP administration showed increased resorption rates of embryos, decreased number and diameter of uterine arterioles, and disturbances of maternal–fetal immune microenvironment, which compromises embryos development [94].

Metabolic disorders were also observed in offspring of dams exposed to PS MP by drinking water during pregnancy [50, 51] and lactation [50]. To evaluate the long-term effects of MP and NP exposure, Luo and colleagues analyzed physiological, pathological, and metabolism indicators of adult F1 offspring (40-weeks old) of dams exposed to PS MP during pregnancy and lactation. Adult female F1 offspring showed increased lipid accumulation in the liver [50]. Furthermore, pregnant mice exposed to MP and NP by IV administration showed decreased embryo body weight, although not affecting the number of embryos [95]. In addition, mice dams exposed to 60 nm NP showed decreased placental diameter and extravasation in fetuses and placenta [95].

#### Neurotoxicity

Nanoplastics can cross the blood–brain barrier in a size-dependent manner [96]. Bioaccumulation, altered lipid peroxidation, and disrupted activity of neurotransmitters have been reported in the brains of marine organisms and fish [96, 97]. However, plastic-mediated neurotoxicity in rodents has been poorly investigated so far [97]. While no significant differences in cognitive function were observed in rats exposed to PS NP for five weeks by drinking water, the authors noted that the small sample size (n = 6) and limited testing unlikely reflected subtle, transient effects [98].

Estrela and colleagues observed impaired object recognition in response to PS NP exposure, coinciding with redox changes, reduced acetylcholinesterase (AChE) activity, and accumulation of particles in the brain [22]. Nonetheless, administration of particles systemically (IP) does not reflect the first-pass effect and is not considered a relevant exposure route for environmental MP and NP. Furthermore, altered neurotransmitter activity following MP and NP accumulation was observed in organs besides the brain, such as the liver [41], highlighting the potential for particles to damage CNS function in multiple tissues. In addition, indirect effects of particle exposure, such as pro-inflammatory mediators from other accumulation sites, may also result in neurotoxicity [99].

#### Other effects

The potential effects of MP and NP exposure in other tissues are still poorly investigated in rodent models. For example, rats exposed daily to MP for 90 days by drinking water showed myocardium alterations, such as vascular congestion, areas with thinner muscle fibers and ruptures, and increased serum heart damage markers (CK-MB and Troponin I) [100]. Also, increased apoptosis and oxidative stress in the heart were observed, which triggered activation of the Wnt/β-catenin signaling pathway related to myocardium fibrosis [100].

Another concern is the potential toxicity in endocrine tissues caused by plastics. For example, rats exposed daily to PS NP for five weeks by oral gavage showed decreased active forms of thyroid hormones (FT3 and FT4) in circulation and increased levels of TSH with high doses of NP, followed by changes in cholesterol serum markers and more liver damage. Hence, PS NP administration could interfere with lipid metabolism by disrupting the thyroid endocrine system [101].

The pathophysiology of chronic inflammatory diseases and co-morbidities of metabolic syndrome may be exacerbated in individuals exposed to excessive MP and NP levels. Administration of 5 µm PS particles by drinking water in a murine acute colitis model enhanced hepatic lipid disruption and intestinal barrier dysfunction [85]. Serum inflammatory markers were higher in mice with colitis than in control animals exposed to MP, indicating the potential for sensitization of individuals with substantial plastic loads to chronic diseases.

#### Future perspectives

New studies are continuously published regarding possible harmful effects in terrestrial mammalian organisms caused by plastic particles. However, most studies have a set of inherent challenges that need to be overcome. Considering plastic particles are found everywhere, the first challenge is the presence of contaminants during analysis. Contaminants were described in detecting plastic particles in controls, possibly from contact with air and plastic released from clothing and laboratory materials. In addition, the high diversity of plastic properties, such as insolubility to non-harmful solvents and buoyancy, can compromise the main experimental models to assess toxicity.

Another challenge is the availability of environmental plastics, like heterogeneous mixtures compared to commercially available plastics used in studies, which cannot be extrapolated to reality. This lack of studies on environmental plastics is mainly related to poor improvement in sampling, processing, and detection of plastics loads, which also compromises estimations of MP and NP doses found in the environment. This issue converges with another challenge: doses applied in many in vivo studies do not correspond to plastics concentrations found in the environment, and studies using environmentally relevant doses showed no effects, diverging from high doses experiments.

Many variables and conditions are applied in different studies designs; thus, considering multiple testing problems that could be related to data and performing proper adjustments for each case is needed for satisfactory conclusions and suggestions. Studies may use the precautionary principle as an argument for evaluating exposure to high doses of MP and NP before assessing the environmental dose. However, literature bias may occur for publications demonstrating effects, conflicting with the studies using low doses, as they might show different results or absence of effects. Furthermore, low incentives for studies with no effects may further compromise a critical debate regarding exposure to MP and NP.

Although plastics are compounds that can be in nature for a long time, longitudinal monitoring for plastic toxicology remains poorly explored. Experimental chronic models assessing only one terminal endpoint may not show effects, hence questioning the exposure period required to observe effects. Additionally, improvement in experimental designs for long-term and chronic studies may help comprehend immunogenic responses to prolonged plastic exposure.

Several knowledge gaps were addressed in this review: synergistic or antagonistic effects of particle mixtures on uptake, biodistribution, bioaccumulation, clearance, and biological responses; standardized method(s) of assessment of particle combinations or environmental plastics is vital for appropriate risk assessment of reliable exposure concentrations and time; lack of non-invasive or non-destructive estimation of particle load and biodistribution at an adequate resolution. These knowledge gaps may be filled by improving sampling, processing, and detection in optimal resolution, leading to better estimations and the development of experimental designs closer to the environment.

## Conclusion

Understanding cytotoxic effects of plastic exposure requires more progress in several fields. First, standardized sampling techniques and improved characterization of environmental MP and NP are needed. Second, will there is a good body of evidence on acute plastic exposure, chronic exposure over longer time frames in higher organisms is understudied. Third, consensus on the effects and methodological tools on the presence of plastic in vertebrates in different types of organs are lacking to better understand potential relationships to chronic inflammation and disease. More research is needed to shed light on those aspects to better understand the consequences of plastic exposure in human health and environmental risks.

## Data Availability

Not applicable.
